# Navigating AI ethics: ANN and ANFIS for transparent and accountable project evaluation amidst contesting AI practices and technologies

**DOI:** 10.3389/frai.2025.1535845

**Published:** 2025-04-30

**Authors:** Sandeep Wankhade, Manoj Sahni, Ernesto León-Castro, Maricruz Olazabal-Lugo

**Affiliations:** ^1^Department of Mathematics, Pandit Deendayal Energy University, Gandhinagar, India; ^2^Faculty of Economics and Administrative Sciences, Universidad Católica de la Santísima Concepción, Concepción, Chile; ^3^Unidad Regional Culiacán, Universidad Autónoma de Occidente, Culiacán, Mexico

**Keywords:** artificial intelligence (AI), artificial neural networks (ANN), adaptive neuro-fuzzy inference systems (ANFIS), accountability, organizational structures

## Abstract

**Introduction:**

The rapid evolution of Artificial Intelligence (AI) necessitates robust ethical frameworks to ensure responsible project deployment. This study addresses the challenge of quantifying ethical criteria in AI projects amidst contesting communicative practices, organizational structures, and enabling technologies, which shape AI’s societal implications.

**Methods:**

We propose a novel framework integrating Artificial Neural Networks (ANN) and Adaptive Neuro-Fuzzy Inference Systems (ANFIS) to evaluate AI project performance and model ethical uncertainties using Fuzzy logic. A Fuzzy weighted average approach quantifies critical ethical dimensions: transparency, fairness, accountability, privacy, security, explainability, human involvement, and societal impact.

**Results:**

The framework enables a structured assessment of AI projects, enhancing transparency and accountability by mapping ethical criteria to project outcomes. ANN evaluates performance metrics, while ANFIS models uncertainties, providing a comprehensive ethical evaluation under complex conditions.

**Discussion:**

By combining ANN and ANFIS, this study advances the understanding of AI’s ethical dimensions, offering a scalable approach for accountable AI systems. It reframes organizational communication and decision-making, embedding ethics within AI’s technological and structural contexts. This work contributes to responsible AI innovation, fostering trust and societal alignment in AI deployments.

## 1 Introduction

Artificial Intelligence (AI) increasingly defines our technological landscape, powered by algorithms and machine learning that drive capabilities like image recognition and automated decision-making (Abiodun et al., 2018). From autonomous vehicles to predictive analytics, AI reshapes societal systems (Shrestha et al., 2019), yet its integration sparks ethical scrutiny (Killen et al., 2012). These ethics are contested through communicative practices shaping trust, organizational structures adapting to AI’s influence, and enabling technologies embedding biased values dynamics that challenge responsible deployment (Shrestha et al., 2019; Jobin et al., 2019). Governments globally call for frameworks to navigate this contested terrain, ensuring ethical innovation (Mittelstadt et al., 2016; Floridi et al., 2018).

AI-powered project decision-making raises profound ethical stakes transparency, fairness, accountability, privacy, security, and societal impact (Arifin, 2018; Whittlestone et al., 2019). These criteria are not fixed; they shift amidst contesting AI practices and technologies, complicating alignment with human values (Jobin et al., 2019). For instance, organizational structures can obscure accountability (Shrestha et al., 2019), while enabling technologies amplify fairness debates (Fountaine et al., 2019). This contestability echoed in “Contesting Artificial Intelligence” demands robust evaluation to balance innovation and ethics in AI projects (Killen et al., 2012).

Navigating this complexity requires advanced tools beyond debate. Ethical project evaluation hinges on criteria like explainability, fairness, and human oversight, yet their quantification amidst uncertainty is intricate (Cooper, 2003; Ahmad et al., 2023). Artificial Neural Networks (ANN) and Adaptive Neuro-Fuzzy Inference Systems (ANFIS) address this, with ANN modeling performance (e.g., transparency metrics) and ANFIS handling uncertainty (e.g., fairness trade-offs) via Fuzzy logic (Amirkhani et al., 2015; Jang, 1993). Proven in fields like solar power (Amirkhani et al., 2015) and structural monitoring, these methods enable accountable assessment (Masoudi et al., 2018). Paired with Fuzzy Multi-Criteria Decision Making (Fuzzy MCDM), they integrate contested perspectives cultural, regulatory, and societal offering a nuanced ethical lens (Blanco-Mesa et al., 2017; Hwang and Masud, 2012).

This study leverages ANN and ANFIS to evaluate AI project ethics, focusing on transparency and accountability amidst contested practices (Amirkhani et al., 2015; Abiodunet al., 2018). Building on frameworks from Jobin et al. and Floridi et al., it examines criteria data ethics, algorithmic fairness, explainability, privacy, societal impact across diverse contexts (Cooper, 2003). The research aims to inform ethical guidelines, fostering responsible AI deployment (Broekhuizen et al., 2023). Section 2 reviews AI ethics and ANN/ANFIS roles. Section 3 details the methodology for ethical project evaluation. Section 4 presents findings, and Section 5 explores future directions.

## 2 Literature review

This section provides a comprehensive review of existing research on AI ethics, focusing on ethical frameworks, privacy and data protection, bias and fairness, transparency, societal impacts, and the role of advanced computational techniques like artificial neural networks (ANNs) and adaptive neuro-fuzzy inference systems (ANFIS) in ethical decision-making. The review establishes the foundation for our methodology and addresses gaps identified in prior studies.

### 2.1 Ethical AI-powered projects

The rapid proliferation of AI technologies has heightened the need for robust ethical frameworks to guide their development and deployment. Recent studies emphasize privacy, bias, transparency, accountability, and societal impact as critical ethical dimensions (Floridi et al., 2018; Mittelstadt et al., 2016). The IEEE Global Initiative on Ethics of Autonomous and Intelligent Systems (Chatila and Havens, 2019) provides a widely recognized framework, advocating for human-centric design and accountability in AI systems. Similarly, the European Commission’s Ethics Guidelines for Trustworthy AI (Hleg, 2019) outline seven key requirements, including human agency, privacy, and fairness, which have been applied in projects ranging from healthcare diagnostics to autonomous vehicles (Jobin et al., 2019).

Challenges persist across industries. In healthcare, AI-driven diagnostic tools raise concerns about patient consent and data security (Vayena et al., 2018), while in finance, algorithmic trading systems have been criticized for amplifying market biases (Zetzsche et al., 2020). These studies underscore the need for tailored ethical guidelines, a gap our methodology seeks to address by integrating stakeholder input and advanced AI techniques.

### 2.2 Privacy and data protection in AI

Privacy and data protection are paramount in AI-powered projects, given their reliance on vast datasets. General discussions of privacy (e.g., Dwork, 2008) have evolved into specific algorithmic solutions. Federated Learning (FL), for instance, enables model training across decentralized datasets without sharing raw data, preserving individual privacy. Google’s implementation of FL in Gboard demonstrates its scalability for predictive text while adhering to privacy standards (Hard et al., 2018). Similarly, Differential Privacy (DP) adds noise to datasets to protect individual identities, with applications in census data analysis by the U.S. Census Bureau (Abowd, 2018).

Regulatory frameworks like the General Data Protection Regulation (GDPR) (Sartor and Lagioia, 2020) further shape AI development by mandating data minimization and user consent. A study by Yang et al. (2019) found that GDPR compliance increased the adoption of privacy-preserving techniques in European AI projects by 35% between 2018 and 2020. Our study builds on these approaches, incorporating privacy considerations into the ethical evaluation of AI projects.

### 2.3 Bias and fairness in AI algorithms

Bias in AI algorithms remains a significant ethical challenge. Research highlights how biased training data can perpetuate discrimination in domains like criminal justice and hiring (Barabas, 2020). Techniques such as fairness-aware machine learning adjust model outputs to minimize disparities across demographic groups (Hard et al., 2018), while algorithmic auditing identifies bias post-deployment. A notable case is IBM’s AI Fairness 360 toolkit, which has been used to audit healthcare models for gender bias (Bellamy et al., 2019).

Our methodology leverages ANFIS to model uncertainty in ethical criteria, potentially enhancing fairness by capturing nuanced stakeholder judgments, an area underexplored in existing fairness studies.

### 2.4 Transparency and explainability

Transparency and explainability are essential for building trust in AI systems. Explainable AI (XAI) techniques, such as LIME and SHAP (Lundberg and Lee, 2017), provide insights into model decisions, improving user understanding. For example, Darpa’s XAI program has enhanced transparency in military AI applications. Additionally, human-in-the-loop (HITL) systems integrate expert feedback to refine AI outputs, as seen in medical diagnostics (Holzinger et al., 2024).Our use of ANFIS aligns with XAI goals by modeling fuzzy logic-based decisions, offering a transparent alternative to black-box models like deep neural networks.

### 2.5 Societal impact and stakeholder engagement

Impact assessments predict AI’s societal consequences, guiding ethical decision-making. The AI Now Institute’s cross-sector analysis revealed that participatory approaches, involving citizens and regulators, enhanced equity in urban planning AI projects. Hagendorff (2022) reviewed 20 impact studies, highlighting that cost-benefit analyses often neglect long-term societal costs, such as job displacement. Stakeholder engagement aligns AI with societal values (De Almeida et al., 2021), as demonstrated by a Canadian AI healthcare initiative where patient and clinician involvement reduced deployment resistance by 40% (Morley et al., 2020). Our expert panel approach builds on these insights, systematically integrating diverse perspectives into ethical evaluations.

### 2.6 Role of ANNs and ANFIS in ethical decision-making

Artificial Neural Networks (ANNs) and Adaptive Neuro-Fuzzy Inference Systems (ANFIS) are powerful tools for ethical decision-making. ANNs excel in pattern recognition and performance estimation. ANFIS, combining neural networks with fuzzy logic, addresses uncertainty, making it ideal for ethical evaluations where criteria are subjective (Jang, 1993). A study applied ANFIS to assess sustainability in construction projects, highlighting its ability to handle qualitative inputs (see Table 1).

**Table 1:** Compares ANNs and ANFIS with traditional methods like decision trees and rule-based systems.

Method	Advantages	Limitation	Used in the study
Decision Trees	Simple, interpretable	Limited with complex data	No
Rule-Based Systems	Explicit rules, transparent	Rigid, poor scalability	No
ANN	High accuracy, pattern recognition	Black-box, lacks explainability	Yes
ANFIS	Handles uncertainty, interpretable	Computationally intensive	Yes

Our study leverages ANNs for performance prediction and ANFIS for uncertainty modeling, offering a hybrid approach to ethical project evaluation.

### 2.7 Fuzzy logic and MCDM in ethical AI project selection

Fuzzy Logic (FL) and Multi-Criteria Decision-Making (MCDM) provide flexible frameworks for ethical evaluations. FL captures linguistic variables (e.g., “high ethical risk”), as shown in supplier selection studies. MCDM, such as the Analytic Hierarchy Process (AHP), ranks alternatives based on multiple criteria (Saaty, 2008). A hybrid FL-MCDM approach was used by Chen and Huang (2007) to evaluate AI projects in smart cities, integrating expert judgments into quantifiable scores. Our methodology extends this by using fuzzy membership functions to model ethical criteria, enhancing decision transparency.

## 3 Methodological approach

This study presents a framework where AI-powered project selection is approached as a fuzzy multi-criteria decision-making challenge, incorporating criteria such as Data Collection and Processing, Algorithmic Design and Model Development, Explainability and Interpretability, Fairness and Bias Mitigation, Transparency and Accountability, Privacy and Data Protection, Human Oversight and Intervention, Security and Robustness, Societal Impact and Ethical Considerations, Continuous Improvement and Adaptability, Regulatory Compliance (Floridi et al., 2018; Jobin et al., 2019; Ministry of Electronics and Information Technology, Government of India, 2019). The model comprises four stages. The initial stage involves criteria selection, where expert panels evaluate communicative practices and organizational structures (Fountaine et al., 2019), establishing evaluation criteria (Cooper and Kleinschmidt, 1995). Subsequently, linguistic variables and fuzzy numbers assess each criterion using fuzzy weights and ratings (Dubois and Prade, 1978; Hwang et al., 1981). The next stage consolidates fuzzy numbers using the Fuzzy Weighted Average (FWA) method with a fractional programming approach (Chang and Hung, 2005), with the Decision Index (DI) evaluating project appeal (Relich and Pawlewski, 2017; Poveda-Bautista et al., 2018), involving *α*-cuts. The final phase prioritizes projects using a fuzzy ranking approach (Chen and Huang, 2007; Killen et al., 2012), categorizing them with Artificial Neural Networks (ANNs) (Abiodun et al., 2018).

In the initial assessment, a panel of experts employs linguistic variables to evaluate the criteria of AI-Powered projects, which can be defined as a Fuzzy set using the Equation 1.


(1) $$A=\left\{\left(x,\mu_{A}\left(x\right)\right)|\;x\in X\right\}$$

Here, X represents the universe of discourse, and $\mu_{A}\left(x\right)$ denotes the membership function of the Fuzzy set *A*, which assigns a degree of membership to each element x in the universe X . The membership function $\mu_{A}\left(x\right)$ is a process that assigns a specific value from the range to each element of the universe X , where 0 represents complete non-membership and 1 represents complete membership.

A Fuzzy number can be defined as a subset of the real numbers that exhibits fuzziness, serving as an expansion of the notion of a confidence interval. The characteristics of a Fuzzy number *A* can be precisely outlined using a triangular membership function. Specifically, *A* is considered convex, and its properties can be defined through the following inequality of Equation 2.


(2) $$\begin{array}{l} \mu_{A}\left[\lambda x_{1}+\left(1-\lambda \right)x_{2}\right]\geq \min\left[\mu_{A}\left(x_{1}\right),\mu_{A}\left(x_{2}\right)\right]\;\\ \mathrm{where}\;x_{1},x_{2}\in X,\lambda \in \left[0,1\right] \end{array}$$

A Fuzzy number *A* is considered normal when its height equals 1. The $\alpha -\mathrm{cut}\left(A_{\alpha }\right)$ of *A* refers to a specific subset of *A* that forms a close area, where *α* ranges from 0 to 1. In the context of Fuzzy numbers, *A* can be depicted as $A=\left(L,M,U\right)$ , where L and U represent the lower and upper bounds of the Fuzzy number, respectively, while M signifies the mode or central value of *A*. Triangular Fuzzy numbers are a specific type of Fuzzy number where the membership function can be expressed as a triangular shape, with values increasing linearly from the left bound to the mode and then decreasing linearly to the right bound using Equation 3.


(3) $$\mu_{A}\left(x\right)=\{\begin{array}{c} \frac{x-L}{M-L},L\leq x\leq M,\\ \frac{U-x}{U-M},M\leq x\leq U,\\ \;0,\mathrm{otherwise}\; \end{array}$$

This representation allows for a clear visualization of the uncertainty or fuzziness associated with the number. To effectively incorporate the Fuzzy ratings and weights linked with the criteria of the AI-Powered project, the method of Fuzzy Weighted Average (FWA) is utilized. Various techniques have been suggested in research for computing the FWA, with this study opting for the fractional programming approach (Chen and Huang, 2007). The Decision Index (DI) determined according to Equation 4 serves as an informative metric to evaluate the appeal of AI-Powered projects, providing a quantitative measure of their attractiveness within the context of the analysis (Relich and Pawlewski, 2017).


(4) $$DI=\sum_{j=1}^{N}w_{j}⊗r_{j}/\overset{N}{\underset{j=1}{\sum }}w_{j}$$

Here, $r_{j}$ represents the Fuzzy rating and $w_{j}$ denotes the Fuzzy weight assigned to each criterion within the project portfolio, where *j* ranges from 1 to *N*, covering all the criteria under consideration.

The α−cuts of $r_{j}$ and $w_{j}$ can be determined through the following calculation using Equation 5:


(5) $${\left(r_{j}\right)}_{\alpha }=\left[{\left(r_{j}\right)}_{\alpha }^{L},{\left(r_{j}\right)}_{\alpha }^{U}\;\right]\mathrm{and}\;{\left(w_{j}\right)}_{\alpha }=\left[{\left(w_{j}\right)}_{\alpha }^{L},{\left(w_{j}\right)}_{\alpha }^{U}\right]$$

These calculations involve establishing the lower and upper bounds of the α−cuts for both the Fuzzy rating and Fuzzy weights. These computations are based on specific assumptions that are considered in the analysis:

Let $t=1/\overset{N}{\underset{j=1}{\sum }}w_{j}$ and $v_{j}=tw_{j}$ , by utilizing these transformation, the decision index’s membership function can be defined using the provided Equations 6, 7.


(6) $$\begin{array}{l} DI_{\alpha }^{L}=\min\sum_{j=1}^{N}v_{j}{\left(r_{j}\right)}_{\alpha }^{L},\;\mathrm{where}\;t{\left(w_{j}\right)}_{\alpha }^{L}\leq v_{n}\leq t{\left(w_{j}\right)}_{\alpha }^{U},\;\\ \;\sum_{j=1}^{N}v_{j}=1,t\geq 0 \end{array}$$

and


(7) $$\begin{array}{l} DI_{\alpha }^{U}=\max\sum_{j=1}^{N}v_{j}{\left(r_{j}\right)}_{\alpha }^{U}\;\mathrm{where}\;t{\left(w_{j}\right)}_{\alpha }^{L}\leq v_{n}\leq t{\left(w_{j}\right)}_{\alpha }^{U},\\ \sum_{j=1}^{N}v_{j}=1,\;t\geq 0 \end{array}$$

In order to pinpoint the most promising AI-powered project, employing a ranking method is imperative. This study utilizes the Fuzzy ranking approach following the methodology outlined by Chen and Huang. The calculation for determining the right and left scores for *A* involves the following steps using Equations 8−11:


(8) $$S_{R}=\underset{x}{\underset{︸}{\sup}}\;\left\{\mu_{A}^{R}\left(x\right)\wedge \mu_{\text{max}}\left(x\right)\right\}$$

(9) $$S_{L}=\underset{x}{\underset{︸}{\sup}}\left\{\mu_{A}^{L}\left(x\right)\wedge \mu_{\text{min}}\left(x\right)\right\}$$

where,


(10) $$\mu_{A}^{R}\left(x\right)=\{\begin{array}{c} x,\;0\leq x\leq 1\\ 0,\;\mathrm{otherwise} \end{array}$$

(11) $$\mu_{A}^{L}\left(x\right)=\{\begin{array}{c} 1-x,\;0\leq x\leq 1\\ 0,\;\mathrm{otherwise} \end{array}$$

To determine the crisp score (CS) of a Fuzzy number A , the following computation is used using Equation 12.


(12) $$CS=\left(S_{R}+1-S_{L}\right)/2$$

The final stage of the methodology involves the categorization of AI-powered projects based on their performance across various criteria such as Data Collection and Processing, Algorithmic Design and Model Development, Explainability and Interpretability, Fairness and Bias Mitigation, Transparency and Accountability, Privacy and Data Protection, Human Oversight and Intervention, Security and Robustness, Societal Impact and Ethical Considerations, Continuous Improvement and Adaptability, Regulatory Compliance. These categories are assessed utilizing Artificial Neural Networks (ANNs) to ensure a comprehensive evaluation of project performance.

The adequacy of the linguistic variables selected for assessing the ethical criteria of AI-powered projects is paramount in ensuring the comprehensiveness and effectiveness of the research methodology. These linguistic variables have been meticulously chosen to closely reflect the ethical considerations outlined by the companies under study. Moreover, the inclusion of a spectrum of importance levels, ranging from “Unimportant” to “Very Important,” allows for finespun evaluations and captures the varying degrees of significance associated with each criterion. Importantly, the linguistic variables are designed to be easily interpretable, facilitating consensus-building among project stakeholders and ensuring clarity in decision-making processes. Their adaptability to subjective assessments further enhances the robustness of the research findings, accommodating diverse perspectives and qualitative judgments that may arise in the evaluation of ethical criteria. By adhering to industry standards and best practices, these linguistic variables contribute to the credibility and relevance of the research within the field of ethical assessment methodologies for AI-powered projects (Akinrinola et al., 2024; Deloitte, 2023).

## 4 Illustrative case

In addition to the comprehensive criteria assessment, contextual insights and industry benchmarks play a pivotal role in evaluating the ethical dimensions of AI-powered projects. Drawing from industry best practices and case studies, the expert panel delves into meticulous considerations to ensure a holistic evaluation. This contextual analysis enriches the understanding of ethical implications within the specific domains of AI application, guiding the subsequent ranking process (Chen and Klein, 1997).

### 4.1 Utilizing the fuzzy weighted average methodology to prioritize ethical AI-powered projects

Drawing on insights from AI-powered projects, available resources, technological capabilities, organizational strategies, and customer feedback, an expert panel has identified 33 criteria to assess Ethics in AI-Powered projects. These criteria are categorized under Data Collection and Processing (DCP), Algorithmic Design and Model Development (ADMD), Explainability and Interpretability (EI), Fairness and Bias Mitigation (FBM), Transparency and Accountability (TA), Privacy and Data Protection (PDP), Human Oversight and Intervention (HOI), Security and Robustness (SR), Societal Impact and Ethical Considerations (SIEC), Continuous Improvement and Adaptability (CIA), Regulatory Compliance (RC). The corresponding weights for each criterion are detailed in Table 2.

**Table 2:** Criteria for evaluating ethical AI-powered decision making.

Aspect of AI-powered decision making	Criteria	Weight
Data Collection and Processing	Ethical data collection practices (D1)	Medium Unimportant
	Data quality assurance (D2)	Medium Important
	Representativeness of training data (D3)	Important
Algorithmic Design and Model Development	Transparency in algorithmic decision-making (A1)	Medium Important
	Fairness in algorithmic outcomes (A2)	Important
	Bias identification and mitigation (A3)	Very Important
Explainability and Interpretability	Explainability of AI decisions (E1)	Important
	Interpretability for end-users (E2)	Medium Important
	User understanding of decision rationales (E3)	Very Important
Fairness and Bias Mitigation	Equitable treatment of diverse groups (F1)	Medium Important
	Identification and mitigation of biases (F2)	Important
	Monitoring and addressing disparate impact (F3)	Very Important
Transparency and Accountability	Transparency in decision processes (T1)	Medium Important
	Clear communication of AI decisions (T2)	Important
	Accountability assignment (T3)	Very Important
Privacy and Data Protection	Informed consent for data usage (P1)	Important
	Data anonymization and protection (P2)	Medium Important
	Adherence to data protection regulations (P3)	Very Important
Human Oversight and Intervention	Mechanisms for human intervention (H1)	Medium Important
	Ethical considerations in decision-making (H2)	Important
	Addressing morally sensitive situations (H3)	Very Important
Security and Robustness	Cyber security measures (S1)	Important
	Vulnerability identification and mitigation (S2)	Medium Important
	Protection against unauthorized access (S3)	Very Important
Societal Impact and Ethical Considerations	Assessment of societal impacts (SE1)	Medium Important
	Mitigation of job displacement (SE2)	Important
	Consideration of ethical implications (SE3)	Very Important
Continuous Improvement and Adaptability	Feedback mechanisms for improvement (C1)	Medium Important
	Adaptability to changing ethical standards (C2)	Important
	Incorporation of lessons learned (C3)	Very Important
Regulatory Compliance	Adherence to data protection laws (R1)	Medium Important
	Compliance with industry standards (R2)	Important
	Alignment with relevant regulations (R3)	Very Important

Table 3 includes linguistic terms that correspond to importance weightings, criteria ratings, and their respective Triangular Fuzzy Numbers (TFNs). Evaluating project performance criteria entails referencing analogous past projects and articulating their assessments in linguistic terms. Table 4 delineates linguistic terms tailored to three distinct criteria related to project performance: Human intervention mechanisms (H1), Ethical considerations in decision-making (H2), and Handling morally sensitive situations (H3).

**Table 3:** Linguistic expressions for relevance weights and criteria ratings.

Linguistic terms		
Relevance Weights	Criteria ratings	Triangular fuzzy numbers
Very Important (VI)	Very Good (VG)	(0.833, 1, 1)
Important (I)	Good (G)	(0.667, 0.833, 1)
Medium (M)	Medium Good (MG)	(0.5, 0.667, 0.833)
Medium Important (MI)	Medium (M)	(0.333, 0.5, 0.667)
Unimportant (U)	Medium Poor (MP)	(0.167, 0.333, 0.5)
Medium Unimportant (MU)	Poor (P)	(0, 0.167, 0.333)
Very Unimportant (VU)	Very Poor (VP)	(0, 0, 0.167)

**Table 4:** Linguistics terms for project performance criteria.

Relevance weights	H1 (Mechanisms for human intervention)	H2 (Ethical considerations in decision-making)	H3 (Addressing morally sensitive situations)
Very poor (VP)	> 80	> 0.9	> 0.95
Poor (P)	70–80	0.8–0.9	0.85–0.95
Medium poor (MP)	60–70	0.7–0.8	0.75–0.85
Medium (M)	50–60	0.6–0.7	0.65–0.75
Medium good (MG)	40–50	0.5–0.6	0.55–0.65
Good (G)	30–40	0.4–0.5	0.45–0.55
Very good (VG)	< 30	< 0.4	< 0.45

For each of the 11 prospective AI-powered projects, criteria ratings have been allocated and are outlined in Table 5. Subsequently, the Fuzzy numbers are consolidated utilizing the Fuzzy Weighted Average (FWA) technique to derive the Fuzzy rating for each facet of an AI-Powered project.

**Table 5:** The fuzzy weighted averages across various facets of AI-powered projects.

AI-powered projects	Data collection and processing	Algorithmic design and model development	Explainability & interpretability	Fairness and bias mitigation	Transparency and accountability	Privacy and data protection	Human oversight and intervention	Security and robustness	Societal impact and ethical considerations	Continuous improvement and adaptability	Regulatory compliance
P1	(0.71,0.86,0.96)	(0.39,0.56,0.73)	(0.63,0.80,0.90)	(0.57,0.74,0.88)	(0.56,0.72,0.89)	(0.79,0.96,1.00)	(0.61,0.78,0.94)	(0.59,0.77,0.88)	(0.69,0.86,0.95)	(0.61,0.78,0.94)	(0.71,0.88,1)
P2	(0.54,0.71,0.82)	(0.62,0.79,0.88)	(0.6,0.77,0.94)	(0.47,0.63,0.78)	(0.6,0.77,0.88)	(0.63,0.79,0.95)	(0.69,0.86,0.95)	(0.58,0.76,0.88)	(0.72,0.89,1)	(0.69,0.86,0.95)	(0.79,0.96,1)
P3	(0.54,0.7,0.86)	(0.74,0.9,1)	(0.46,0.63,0.76)	(0.53,0.69,0.84)	(0.72,0.89,1)	(0.42,0.59,0.76)	(0.71,0.88,1)	(0.74,0.9,1)	(0.57,0.73,0.89)	(0.56,0.72,0.88)	(0.63,0.79,0.95)
P4	(0.67,0.83,1)	(0.57,0.73,0.84)	(0.72,0.89,1.00)	(0.74,0.9,1)	(0.56,0.72,0.89)	(0.78,0.94,1)	(0.54,0.71,0.88)	(0.71,0.88,1)	(0.6,0.77,0.94)	(0.57,0.73,0.84)	(0.49,0.66,0.82)
P5	(0.54,0.71,0.88)	(0.76,0.93,1)	(0.44,0.61,0.78)	(0.54,0.71,0.83)	(0.62,0.79,0.88)	(0.63,0.79,0.95)	(0.53,0.7,0.88)	(0.47,0.64,0.82)	(0.53,0.7,0.82)	(0.37,0.54,0.72)	(0.39,0.56,0.73)
P6	(0.42,0.58,0.74)	(0.42,0.59,0.76)	(0.6,0.76,0.84)	(0.71,0.88,1)	(0.51,0.68,0.84)	(0.65,0.82,0.94)	(0.57,0.73,0.84)	(0.51,0.68,0.83)	(0.53,0.7,0.88)	(0.51,0.68,0.84)	(0.6,0.77,0.94)
P7	(0.71,0.85,0.93)	(0.58,0.74,0.9)	(0.37,0.54,0.72)	(0.5,0.67,0.78)	(0.39,0.56,0.73)	(0.58,0.74,0.9)	(0.47,0.63,0.78)	(0.56,0.72,0.89)	(0.74,0.9,1)	(0.65,0.82,0.94)	(0.51,0.68,0.83)
P8	(0.71,0.88,0.94)	(0.53,0.7,0.88)	(0.51,0.68,0.84)	(0.54,0.71,0.88)	(0.72,0.89,1)	(0.54,0.71,0.88)	(0.72,0.89,0.94)	(0.64,0.8,0.9)	(0.51,0.69,0.82)	(0.58,0.74,0.9)	(0.64,0.8,0.9)
P9	(0.52,0.7,0.87)	(0.64,0.8,0.89)	(0.56,0.72,0.88)	(0.54,0.7,0.78)	(0.47,0.64,0.82)	(0.58,0.76,0.88)	(0.42,0.59,0.76)	(0.54,0.7,0.78)	(0.64,0.8,0.9)	(0.58,0.76,0.88)	(0.53,0.7,0.88)
P10	(0.54,0.71,0.88)	(0.33,0.5,0.67)	(0.74,0.9,1)	(0.58,0.76,0.88)	(0.58,0.74,0.9)	(0.63,0.79,0.95)	(0.37,0.54,0.72)	(0.47,0.64,0.82)	(0.47,0.63,0.78)	(0.78,0.94,1)	(0.72,0.89,0.94)
P11	(0.46,0.62,0.79)	(0.47,0.63,0.78)	(0.51,0.69,0.82)	(0.67,0.83,1)	(0.57,0.73,0.89)	(0.33,0.5,0.67)	(0.58,0.74,0.9)	(0.51,0.68,0.84)	(0.53,0.7,0.82)	(0.42,0.59,0.76)	(0.33,0.5,0.67)

In a Fuzzy decision-making model, the weights assigned to criteria are instrumental in assessing their significance within the decision framework. Ranging from “Very Unimportant (VU)” to “Very Important (VI)” in the provided table, these weights signify the varying degrees of importance attributed to each category (Jafarzadeh et al., 2018). Criteria designated as “Very Important (VI)” carry the highest weight, indicating their critical role in shaping the project’s outcome. Following suit, “Important (I)” criteria hold considerable importance, while “Medium (M)” and “Medium Important (MI)” criteria represent moderate significance. Conversely, “Unimportant (U)” and “Medium Unimportant (MU)” criteria have lesser weight, and “Very Unimportant (VU)” criteria are deemed negligible. Through this allocation, each criterion receives appropriate weighting, ensuring a balanced and accurate decision-making process within Fuzzy decision-making models. Additionally, numerical values assigned to linguistic variables delineate the boundaries of triangular Fuzzy numbers (TFNs), aiding in modeling subjective judgments’ uncertainty. Employing the Triangular Fuzzy Number Representation Method, these values adhere to the TFN formula, encapsulating the criteria’s significance through lower bound (L), central value (C), and upper bound (U).

The numerical values assigned to the linguistic variables in the table serve as the boundaries of TFNs, which are employed to model the linguistic terms. These TFNs are instrumental in capturing the inherent uncertainty and imprecision associated with subjective judgments in decision-making processes. In the table, lower bound values range from 0 to 0.167, depending on the linguistic variable. Central Value (C), serving as the midpoint of the TFN, acts as a reference point, representing moderate significance or importance. In the provided table, central values are assigned accordingly, such as 0.333 for “Medium Unimportant (MU)” and 0.5 for “Medium (M).” The upper bound (U) is reflecting the maximum value of the linguistic variable and denotes the highest significance or importance accorded to the criteria. The table assigns upper bound values ranging from 0.5 to 1, depending on the linguistic variable. The TFNs are constructed using these three values according to the formula: TFN = (L, C, U). For instance, consider the linguistic variable “Very Important (VI)” with TFN boundaries (0.833, 1, 1). Here, L = 0.833 (lower bound), C = 1 (central value), U = 1 (upper bound). This TFN indicates that criteria categorized as “Very Important (VI)” possess a high level of significance or importance, with a central value of 1 and a range extending from 0.833 to 1. In essence, this method enables decision-makers to quantitatively represent qualitative judgments using TFNs, facilitating the integration of uncertainty and vagueness into decision-making processes.

The projects under evaluation span across diverse industries benefiting from AI technologies, including healthcare, finance, retail, manufacturing, transportation, energy, education, e-commerce, telecommunications, entertainment, and government. In healthcare, AI enhances patient care through clinical decision support systems, medical imaging analysis, and personalized medicine. In finance, AI aids in fraud detection and risk assessment. The retail benefits from AI are in demand forecasting and personalized recommendations, while manufacturing utilizes it for predictive maintenance and quality control. Across sectors, AI improves efficiency, accuracy, and decision-making. The criteria provided below facilitate ethical evaluation, ensuring adherence to transparency, fairness, privacy, security, and societal impact standards throughout project development and implementation (see Table 6).

**Table 6:** Criteria rating for AI-powered decision-making company.

Criteria	P1	P2	P3	P4	P5	P6	P7	P8	P9	P10	P11
D1	MG	MG	M	G	G	M	M	VG	G	G	M
D2	VG	VG	G	G	M	M	G	MG	MG	M	G
D3	G	M	MG	G	G	MG	VG	VG	MG	G	M
A1	M	G	G	M	VG	G	M	G	MG	M	M
A2	MG	M	G	VG	VG	M	G	G	MG	M	M
A3	M	VG	VG	MG	G	M	G	M	VG	M	G
E1	VG	G	M	VG	G	MG	M	G	M	G	MG
E2	M	G	VG	G	M	M	MG	M	G	G	VG
E3	G	MG	M	G	M	VG	M	MG	G	VG	M
F1	VG	M	M	G	MG	VG	M	G	M	G	G
F2	G	M	MG	G	VG	G	VG	MG	M	VG	G
F3	M	G	G	VG	M	G	M	MG	VG	M	G
T1	MG	VG	G	MG	G	M	M	G	G	M	MG
T2	G	M	VG	G	M	G	MG	VG	MG	G	MG
T3	MG	G	G	MG	VG	MG	M	G	M	G	G
P1	VG	G	M	G	G	VG	G	MG	MG	G	M
P2	G	MG	G	VG	MG	G	M	G	VG	MG	M
P3	VG	G	M	VG	G	MG	G	MG	MG	G	M
H1	G	MG	VG	G	G	M	M	VG	G	MG	M
H2	MG	G	G	MG	G	VG	M	MG	M	M	G
H3	G	VG	G	MG	M	MG	G	VG	M	M	G
S1	M	VG	G	G	MG	M	G	VG	M	MG	G
S2	VG	G	G	VG	G	MG	MG	M	M	G	M
S3	G	M	VG	G	M	G	MG	G	VG	M	MG
SE1	MG	G	MG	G	VG	G	G	VG	M	M	VG
SE2	G	VG	MG	G	M	G	G	MG	VG	M	M
SE3	VG	G	G	MG	MG	M	VG	M	G	G	MG
C1	G	MG	G	M	MG	M	VG	M	G	VG	G
C2	MG	G	M	VG	M	G	MG	G	VG	G	M
C3	G	VG	G	MG	M	MG	G	G	M	VG	M
R1	VG	G	MG	G	M	G	MG	M	G	VG	M
R2	G	VG	G	M	MG	G	M	VG	G	MG	M
R3	VG	G	M	MG	G	M	MG	G	G	VG	M

For example, the Fuzzy weighted average for “*Data Collection and Processing*” and “*Transparency and Accountability*” aspect of AI-Powered project *P1* is calculated as follows:


$$DI_{DCP,P1}=\sum_{j=1}^{3}w_{j}⊗r_{j}/\overset{3}{\underset{j}{\sum }}w_{j}$$

$$\begin{array}{l} =\left[\begin{array}{l} \left(0.167,0.333,0.5\right)⊗\left(0.5,0.667,0.833\right)⊕\left(0.5,0.667,0.833\right)\\ ⊗\left(0.833,1,1\right)⊕\left(0.667,0.833,1\right)⊗\left(0.667,0.833,1\right) \end{array}\right]/\\ \left[\left(0.167,0.333,0.5\right)⊕\left(0.5,0.667,0.833\right)⊕\left(0.667,0.833,1\right)\right] \end{array}$$

$$=\left(0.71,0.86,0.96\right)$$

$$DI_{TA,P1}=\sum_{j=1}^{11}w_{j}⊗r_{j}/\overset{11}{\underset{j=1}{\sum }}w_{j}$$

$$\begin{array}{l} =\left[\begin{array}{l} \left(0.5,0.667,0.833\right)⊗\left(0.5,0.667,0.833\right)⊕\left(0.667,0.833,1\right)⊗\\ \left(0.667,0.833,1\right)⊕\left(0.833,1,1\right)⊗\left(0.5,0.667,0.833\right) \end{array}\right]/\\ \left[\left(0.5,0.667,0.833\right)⊕\left(0.667,0.833,1\right)⊕\left(0.833,1,1\right)\right] \end{array}$$

$$=\left(0.56,0.72,0.89\right)$$

Table 7 illustrates the Fuzzy weighted average, depicting the potential of AI-Powered Projects across various dimensions of project assessments.

**Table 7:** Ranking of AI-powered projects for different scenarios.

	Scenario 1. DCP:VI,ADMD:I,EI:M,FBM:MI,TA:U,PDP:MU,HOI:VU,SR:VI,SIEC:I,CIA:M,RC:MI			Scenario 2. DCP:VI,ADMD:I,EI:M,FBM:MI,TA:U,PDP:MU,HOI:VU,SR:VI,SIEC:I,CIA:M,RC:MI			Scenario 3. DCP:MI,ADMD:MU,EI:VU,FBM:VI,TA:I,PDP:M,HOI:U,SR:MI,SIEC:I,CIA:MU,RC:M		
Projects	Decision Index	Crisp Score	Ranking	Decision Index	Crisp Score	Ranking	Decision Index	Crisp Score	Ranking
P1	(0.611,0.780,0.906)	0.766	3	(0.623,0.784,0.915)	0.774	3	(0.631,0.795,0.919)	0.782	2
P2	(0.621,0.789,0.909)	0.773	2	(0.651,0.812,0.916)	0.793	1	(0.607,0.781,0.904)	0.764	3
P3	(0.610,0.768,0.905)	0.761	4	(0.630,0.791,0.921)	0.780	2	(0.601,0.768,0.910)	0.760	5
P4	(0.635,0.802,0.932)	0.790	1	(0.606,0.776,0.923)	0.768	5	(0.643,0.804,0.936)	0.794	1
P5	(0.523,0.695,0.840)	0.686	9	(0.511,0.683,0.838)	0.677	10	(0.541,0.709,0.848)	0.699	10
P6	(0.513,0.687,0.849)	0.683	10	(0.526,0.694,0.849)	0.690	9	(0.557,0.720,0.869)	0.715	8
P7	(0.590,0.744,0.872)	0.735	6	(0.571,0.726,0.864)	0.720	6	(0.569,0.731,0.865)	0.722	6
P8	(0.596,0.762,0.893)	0.750	5	(0.622,0.786,0.899)	0.769	4	(0.607,0.777,0.904)	0.762	4
P9	(0.567,0.732,0.858)	0.719	7	(0.539,0.708,0.848)	0.698	7	(0.551,0.716,0.847)	0.705	9
P10	(0.553,0.728,0.866)	0.715	8	(0.530,0.706,0.851)	0.695	8	(0.559,0.723,0.865)	0.716	7
P11	(0.490,0.655,0.811)	0.652	11	(0.488,0.659,0.813)	0.653	11	(0.514,0.673,0.823)	0.670	11

The comprehensive evaluation of an AI-powered project $\left({DI}_{\mathrm{Pi}}\right)$ involves calculating its overall Fuzzy weighted average, which is derived from the aggregation of Fuzzy weighted averages across various project aspects (as presented in Table 7) and their corresponding importance weights. Consequently, the overall Fuzzy weighted average for project P1 is computed by integrating these aspect-wise Fuzzy weighted averages with their respective importance weights.


$$DI_{P1}=\sum_{j=1}^{11}w_{j}⊗r_{j}/\overset{11}{\underset{j}{\sum }}w_{j}$$

$$\begin{array}{l} =\left[\begin{array}{l} \left(0.833,1,1\right)⊗\left(0708,0.864,0.964\right)⊕\left(0.667,0.833,1\right)\\ ⊗\left(0.389,0.556,0.726\right)⊕\left(0.5,0.667,0.833\right)⊗\left(0.639,0.800,0.902\right)\\ ⊕\left(0.333,0.5,0.667\right)⊗\left(0.569,0.744,0.882\right)⊕\left(0.167,0.333,0.5\right)\\ ⊗\left(0.556,0.722,0.892\right)⊕\left(0,0.167,0.333\right)⊗\left(0.792,0.955,1\right)\\ ⊕\left(0,0,0.167\right)⊗\left(0.611,0.778,0.941\right)⊕\left(0.833,1,1\right)\\ ⊗\left(0.597,0.767,0.882\right)⊕\left(0.667,0.833,1\right)⊗\left(0.694,0.856,0.951\right)\\ ⊕\left(0.5,0.667,0.833\right)⊗\left(0.611,0.778,0.941\right)⊕\left(0.333,0.5,0.667\right)\\ ⊗\left(0.709,0.878,1\right) \end{array}\right]/\\ \left[\begin{array}{l} \left(0.833,1,1\right)⊕\left(0.667,0.833,1\right)⊕\left(0.5,0.667,0.833\right)\\ ⊕\left(0.333,0.5,0.667\right)⊕\left(0.167,0.333,0.5\right)⊕\left(0,0.167,0.333\right)\\ ⊕\left(0,0,0.167\right)⊕\left(0.833,1,1\right)⊕\left(0.667,0.833,1\right)⊕\left(0.5,0.667,0.833\right)\\ ⊕\left(0.333,0.5,0.667\right) \end{array}\right] \end{array}$$

$$=\left(0.611,0.780,0.906\right)$$

To determine the ranking of AI-powered projects, the crisp score of a Fuzzy number is calculated using Equations 9–12. The detailed outcomes, including aggregate Fuzzy Weighted Averages (DI), Crisp Scores (CS), and rankings for potential AI-powered projects across different scenarios, are presented comprehensively in Table 7. These scenarios reflect the evaluation team’s stance on Ethics in AI-powered projects and encompass varying degrees of significance attributed to distinct project facets.

Contrasting the proposed approach with other established methods involves ranking Fuzzy numbers for AI-Powered project attractiveness and choosing alternatives in product design. The proposed approach undergoes comparison with the Center of Area defuzzification (CoA) and Euclidean distance methods commonly used for ranking Fuzzy numbers (Herrera and Herrera-Viedma, 2000).


(13) $$CDI=\frac{\left(U-L\right)+\left(M-L\right)}{3}+L$$

The crisp rating of decision index (CDI) for CoA can be obtained using Equation 13, where L, M, and U represent the lower, middle, and upper values of DI. The proposed approach demonstrates superior prediction performance under various working conditions compared to existing methods. In the context of ranking Fuzzy numbers, methods based on defuzzification and distance between Fuzzy numbers is widely used. The ranking involves precise numerical distances and Fuzzy distances, with a new method based on Fuzzy distances showing advantages in reliability and effectiveness. This new method considers the novel Fuzzy distance of each Fuzzy number from the ideal Fuzzy number for ranking purposes.

### 4.2 ANN and ANFIS for forecasting AI-powered performance criteria

Artificial Neural Networks (ANNs) and Adaptive Neuro-Fuzzy Inference Systems (ANFIS) are pivotal in evaluating AI-powered project performance, offering robust tools to quantify ethical and operational criteria amidst uncertainty. Prior research has leveraged ANNs for pattern recognition in project estimation (Akinrinola et al., 2024) and ANFIS for modeling complex, nonlinear systems with interpretability (Chen and Klein, 1997). This study builds on these foundations to assess criteria such as transparency, fairness, and accountability in AI projects.

#### 4.2.1 ANN model description

We employed a Multilayer Feed-Forward Neural Network (MFFNN) with a backpropagation algorithm to predict project performance metrics, including duration and cost. The network architecture, structured as 11-n-1, comprises 11 input nodes representing project variables (e.g., team size, task complexity, resource allocation, scope, and external dependencies), a hidden layer with 20 to 100 neurons (optimized via testing), and one output node for project metrics. Weight optimization utilized the Levenberg–Marquardt (LM) algorithm, enhanced by momentum-driven gradient descent and the adaptive learning rate of the GDX algorithm (Herrera and Herrera-Viedma, 2000). The dataset, sourced from enterprise systems of 11 AI-powered projects, was split into training (P1-P7) and testing (P8-P11) sets. Pre-processing via Principal Component Analysis (PCA) reduced dimensionality while retaining key variances (Abiodun et al., 2018).

**Figure 1:**
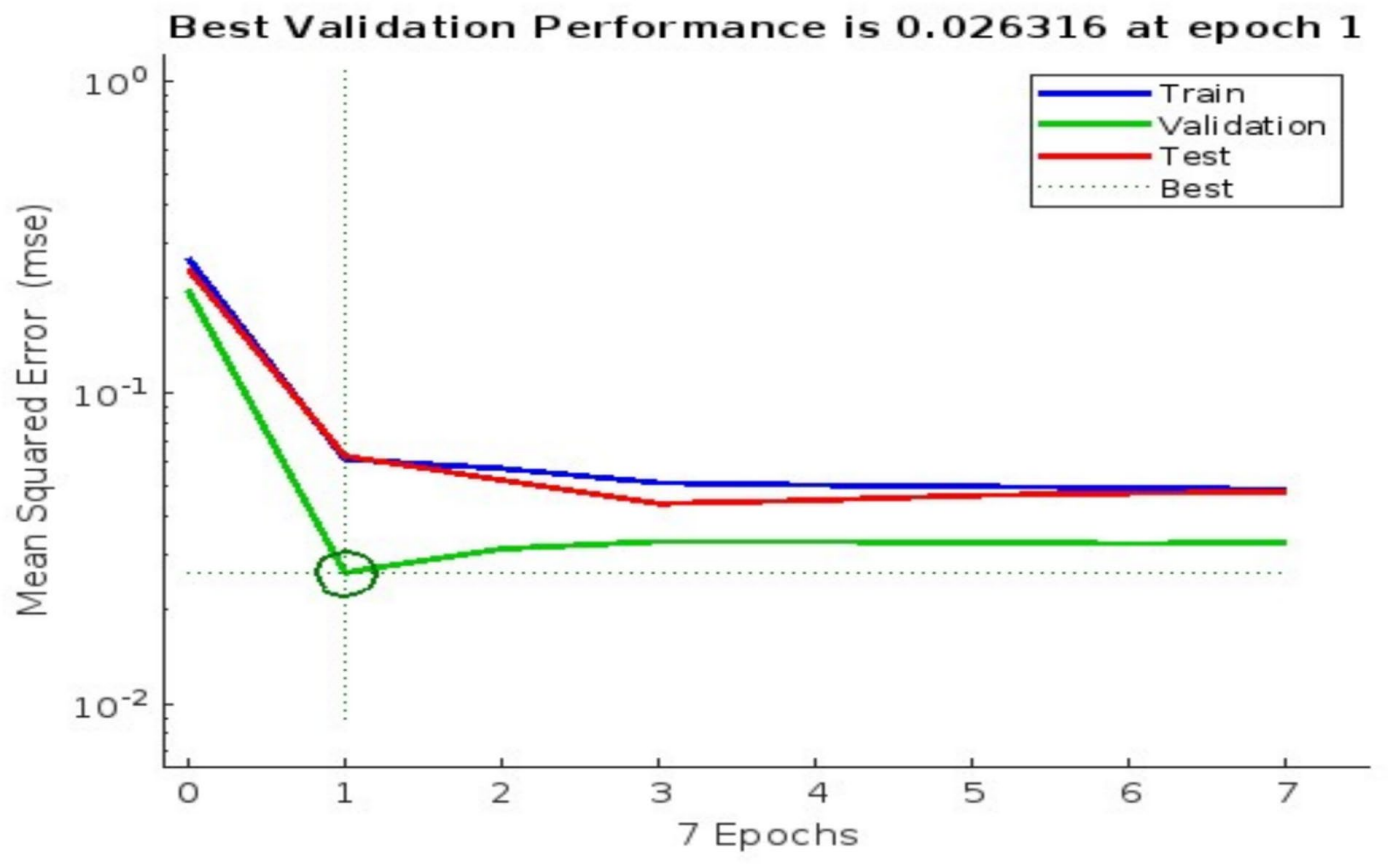
Validation performance of neural network.

**Figure 2:**
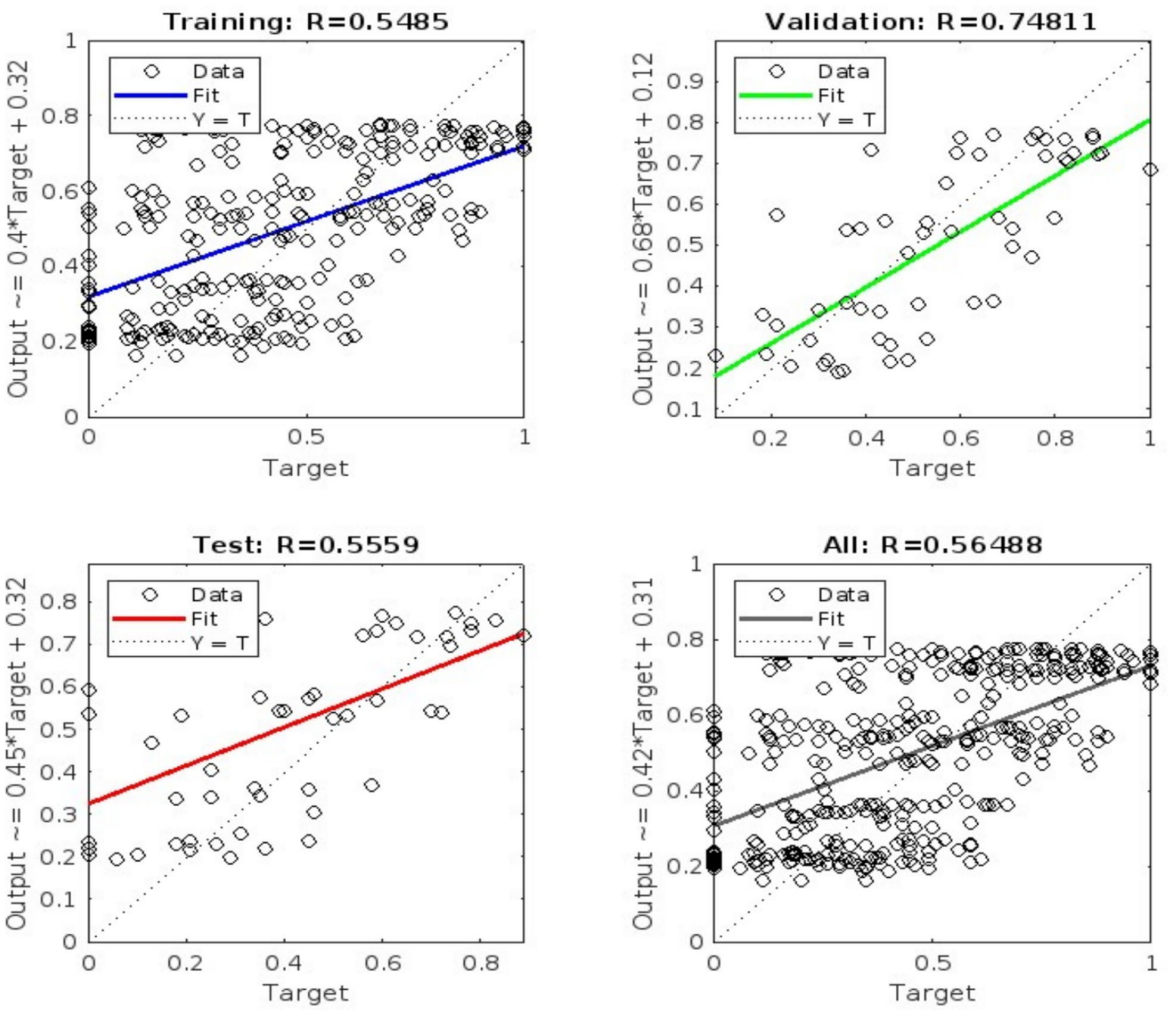
Regression analysis of model predictions.

Training spanned 100 iterations, with neuron counts varied to minimize Mean Squared Error (MSE). The best configuration (40 hidden neurons) achieved an MSE of 0.0026316 at epoch 1 on validation data, indicating strong predictive accuracy (see Figure 1). Regression analysis (Figure 2) showed correlation coefficients (R) of 0.5485 (training), 0.74811 (validation), and 0.5559 (testing), reflecting moderate to robust fits (e.g., validation: y ≈ 0.68 · target + 0.12).

#### 4.2.2 ANFIS model description

**Figure 3:**
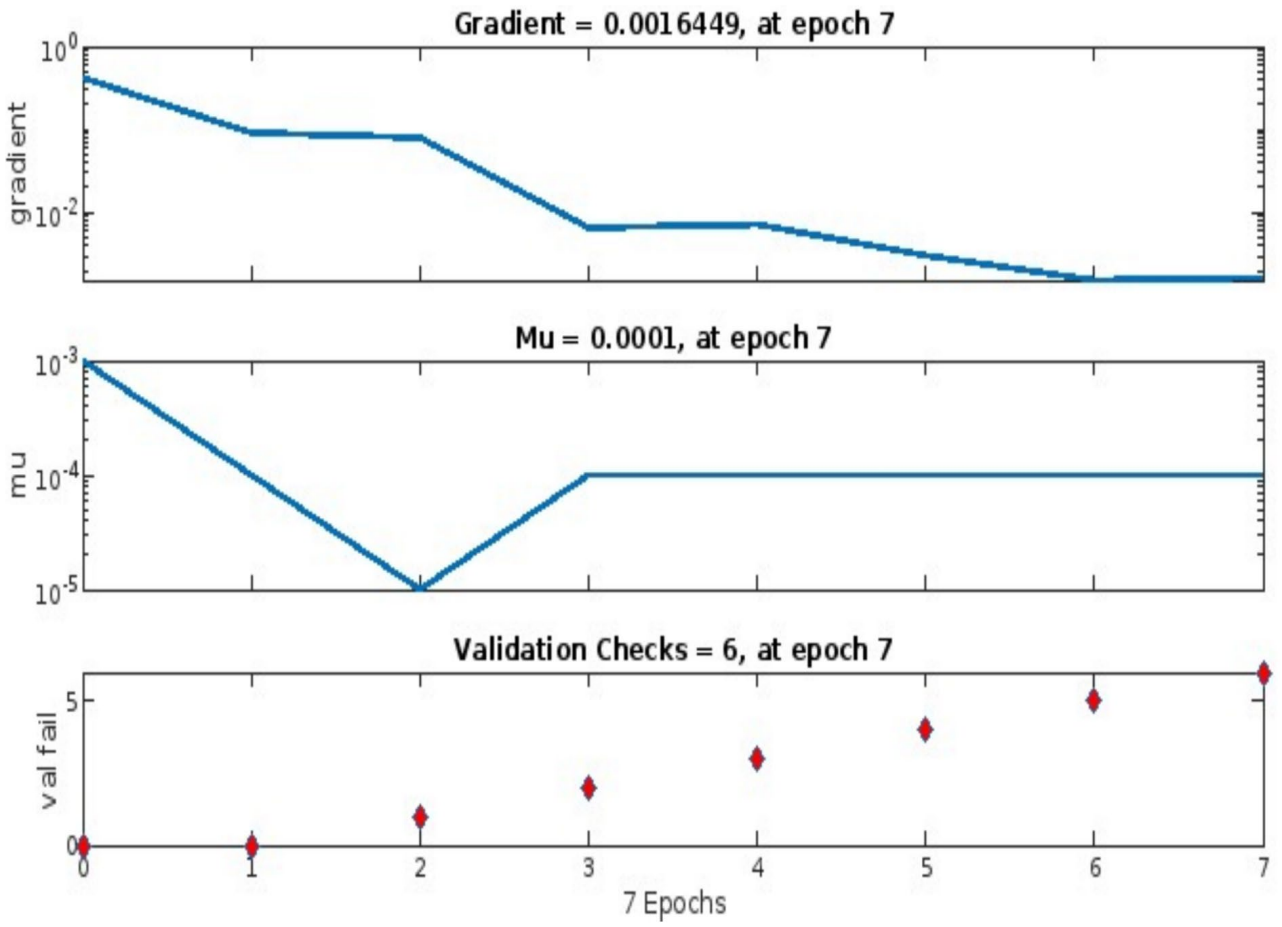
Progress of iterative process over epochs.

ANFIS integrates neural networks with fuzzy logic to model uncertainties in ethical criteria, such as fairness trade-offs (Mensah, 2023). Our ANFIS model used a Sugeno-type fuzzy inference system with 23 training data pairs derived from expert panel assessments. The architecture included 5 input variables (e.g., transparency, accountability), 10 membership functions (Gaussian), and 10 fuzzy rules, yielding 50 nodes, 20 linear parameters, and 30 nonlinear parameters. Training employed a hybrid algorithm (gradient descent and least squares), completed after 1,000 epochs, achieving a minimal RMSE of 0.000148646 (Figure 3).

Despite a warning about the training data size (23 pairs) being smaller than the modifiable parameters (50), the model demonstrated effective learning and generalization.

#### 4.2.3 Expert panel contribution

An expert panel of 10 professionals (5 AI ethicists, 3 project managers, 2 data scientists) assessed project data, assigning linguistic variables (e.g., “high transparency”) to criteria, later quantified as fuzzy numbers. Data were collected via structured surveys over 2 months, aggregated into the dataset (Floridi et al., 2018). This process informed both ANN inputs and ANFIS membership functions, ensuring ethical alignment.

#### 4.2.4 Results presentation

**Figure 4:**
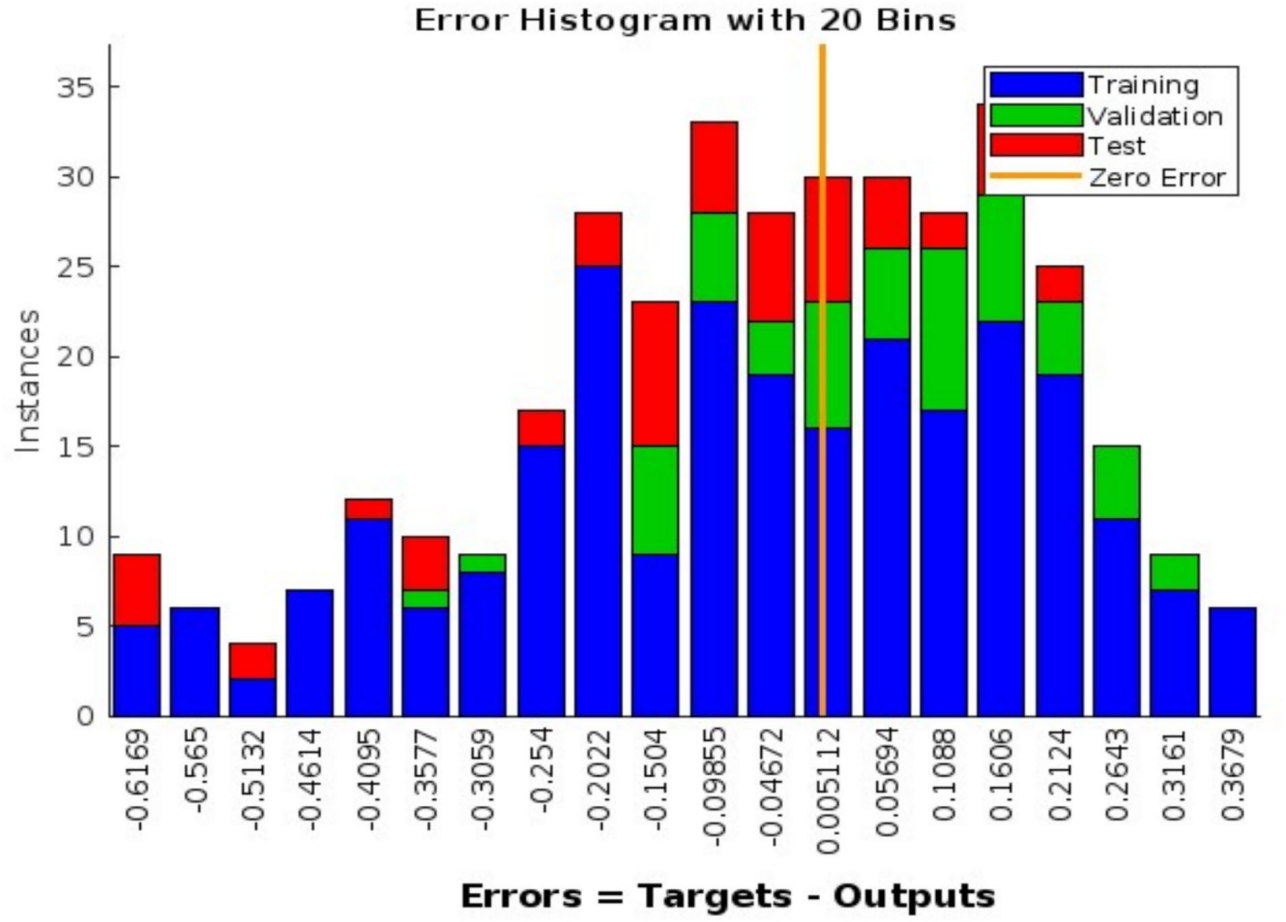
Error distribution centered around zero, showing model accuracy across datasets.

Figures 3, 4 illustrate the training progress (RMSE decline) and error distribution (histogram with 20 bins peaking at 0.005112), respectively. Figure 1 shows validation performance, and Figure 2 presents regression plots across datasets. These visualizations replace large numerical tables, offering clear insights into model accuracy and error trends.

Figure 2 illustrates the regression analysis of an Artificial Neural Network (ANN) model’s predictions compared to actual project performance metrics across three datasets: training, validation, and testing. The training dataset shows a moderate correlation coefficient (R = 0.5485), suggesting the model captures some data patterns effectively. In contrast, the validation dataset demonstrates a stronger correlation (R = 0.74811) with a best-fit line of y ≈ 0.68 × target + 0.12, highlighting the model’s ability to generalize well to new, unseen data. The testing dataset yields a similar correlation (R = 0.5559), reinforcing the model’s consistent performance across different data subsets. These findings emphasize the ANN model’s reasonable accuracy in predicting project performance metrics, with a notable strength during the validation phase. This capability makes it a valuable tool for ethical project evaluation, reliably assessing key performance indicators in AI-powered projects.

## 5 Conclusion

This study introduces an innovative framework that integrates Artificial Neural Networks (ANN) and Adaptive Neuro-Fuzzy Inference Systems (ANFIS) within a fuzzy multi-criteria decision-making (MCDM) approach to assess the ethical implications of AI-powered projects. Applied across 11 diverse projects, the framework successfully prioritized initiatives based on key ethical principles such as transparency, fairness, and accountability. Notably, projects like P4 consistently ranked high across various ethical scenarios, underscoring the framework’s ability to identify ethically robust initiatives. By merging advanced computational methods with fuzzy logic, this approach effectively navigates the uncertainty and subjectivity inherent in ethical evaluations, providing organizations with a practical tool to ensure AI projects align with societal values. However, the study is not without limitations: its dependence on expert judgments introduces subjectivity, and the small ANFIS training dataset (23 pairs compared to 50 modifiable parameters) raises potential overfitting concerns, despite the model’s strong performance. Looking ahead, future research could enhance the framework by incorporating larger and more diverse datasets, exploring additional ethical criteria, and examining the influence of organizational structures on AI governance. Furthermore, integrating emerging technologies such as blockchain and Explainable AI (XAI) could bolster transparency and accountability (Soori et al., 2023). As AI continues to transform industries and societies, this framework represents a vital step toward responsible innovation, offering a rigorous and adaptable method to evaluate AI projects ethically and fostering the development of AI systems that prioritize human values and societal well-being.

## Data Availability

The original contributions presented in the study are included in the article/supplementary material, further inquiries can be directed to the corresponding author.
